# Frequency of *BRCA1* and *BRCA2* causative founder variants in ovarian cancer patients in South-East Poland

**DOI:** 10.1186/s13053-018-0089-x

**Published:** 2018-02-27

**Authors:** Tomasz Kluz, Andrzej Jasiewicz, Elżbieta Marczyk, Robert Jach, Anna Jakubowska, Jan Lubiński, Steven A. Narod, Jacek Gronwald

**Affiliations:** 1 Department of Obstetrics and Gynecology, Fryderyk Chopin University Hospital No 1, Faculty of Medicine of Rzeszow University, 2 Szopena St, 35-055 Rzeszów, Poland; 2 Subcarpatian Oncological Hospital, Genetic Counseling Center, 18 Bielawskiego St, 36-200 Brzozów, Poland; 3 Department of Oncological Surgery, Regional Oncology Center, 11 Garncarska St, 31-115 Kraków, Poland; 4 Deptartment of Gynecology and Obstetetrics, Medical College, Jagiellonian University, 23 Kopernika St, 31-501 Krakow, Poland; 5 Department of Genetics and Pathology, International Hereditary Cancer Center, Pomeranian Medical University, Połabska 4 St, 70-115 Szczecin, Poland; 6 Women’s College Research Institute, 76 Grenville St, Toronto, ON M5G 1N8 Canada

**Keywords:** *BRCA1* and *BRCA2* mutation, Ovarian cancer, Poland

## Abstract

**Background:**

Causative variants in *BRCA1* and *BRCA2* are well-established risk factors for breast and ovarian cancer. In Poland, the causative founder variants in the *BRCA1* are responsible for a significant proportion of ovarian cancer cases, however, regional differences in the frequencies of various mutations may exist. The spectrum and frequency of *BRCA1/2* mutations between ovarian cancer patients have not yet been studied in the region of South-East Poland.

**Methods:**

We examined 158 consecutive unselected cases of ovarian cancer patients from the region of Podkarpacie. We studied 13 Polish causative founder variants in *BRCA1* (c.5266dupC, c.4035delA, c.5251C > T, c.181 T > G, c.676delT, c.68_69delAG, c.3700_3704delGTAAA, c.1687C > T, c.3756_3759delGTCT) and in *BRCA2* (c.658_659delGT, c.7910_7914delCCTTT, c.3847_3848delGT, c.5946delT).

**Results:**

A *BRCA1* causative founder variants were detected in 10 of the 158 (6.3%) ovarian cancer cases. *BRCA2* causative founder variants were not observed. The c.5266dupC mutation was detected in 6 patients, c.181 T > G mutation in 3 patients and the c.676delT mutation in 1 patient. The median age of diagnosis of the 10 hereditary ovarian cancers was 55.5 years of age.

**Conclusions:**

The frequency of 13 causative founder variants in Podkarpacie was lower than in other regions of Poland. Testing of three *BRCA1* mutations (c.5266dupC, c.181 T > G, c.676delT) should be considered a sensitive test panel.

## Background

There are approximately 3600 new ovarian cancer cases and over 2000 deaths as a result of this cancer in Poland annually [1]. It has been shown that ovarian cancer patients from Poland are characterized by a high proportion of a limited number of recurrent mutations in *BRCA1* [2–7]. Three *BRCA1* causative founder variants (c.5266dupC, c.181 T > G, and c.4035delA) account for approximately 80% of all detectable *BRCA1* and *BRCA2* mutations in breast-ovarian cancer families in Poland [2, 3]. A high proportion of *BRCA*-carriers with a limited number of recurrent mutations have an impact on the test costs reduction, it enhances the availability and testing effectiveness. In 2003, Menkiszak et al. observed that 13.5% of ovarian cancer patients in the Szczecin region carry one of these three common causative founder variants in *BRCA1* [8]. Since 2003, several other less frequent recurrent *BRCA1* and *BRCA2* mutations have been reported as well as some regional differences in mutation frequency and spectrum have been observed [2–8]. Currently, five *BRCA1* causative founder variants (c.5266dupC, c.181 T > G, and c.4035delA, c.68_69delAG, c.3700_3704delGTAAA) are recommended in national screening program.

The aim of this study was to evaluate the prevalence and spectrum of 13 *BRCA1* and *BRCA2* causative founder variants in unselected patients diagnosed with ovarian cancer from Region of Podkarpacie. These observations may be important to optimize the genetic testing strategy for ovarian cancer patients in the region.

## Methods

The ovarian cancer cases were identified among clinical base patients of the Department of Obstetrics and Gynecology of Fryderyk Chopin University Hospital No 1 in Rzeszów, Poland between January 2013 and January 2017. All patients were inhabitants of the South-East region of Poland. The study group consisted of 158 consecutive, newly diagnosed cases of ovarian cancer after surgical treatment, unselected based on age or family history. The mean age of diagnosis was 58.5 years (range 22–84 years). The reference pathologist reviewed a representative slide from each cancer to confirm the diagnosis. 20% of patients were diagnosed in I and II clinical stage and 80% in III and IV according to FIGO. 14.8% of ovarian cancers showed pathological grading G1, 11.1% - G2, and 74.1% – G3. A cancer family history was obtained during an appointment with clinical geneticist.

DNA was isolated from 5 to 10 ml of blood. All women were tested for the presence of 13 causative founder variants in *BRCA1* (nine mutations) and *BRCA2* (four mutations). The c.4035delA and c.5266dupC mutations were detected using allele-specific oligonucleotide polymerase chain reaction (PCR) [7]. The other mutations of *BRCA1* (c.5251C > T, c.181 T > G, c.676delT, c.68_69delAG, c.3700_3704delGTAAA, c.1687C > T, c.3756_3759delGTCT) and in *BRCA2* (c.658_659delGT, c.7910_7914delCCTTT, c.3847_3848delGT, c.5946delT) were genotyped using TaqMan assays (Applied Biosystems/Life Technologies, Carlsbad, CA) on Roche LightCycler 480. All mutations were confirmed by Sanger direct sequencing. Sequencing reactions were performed using a BigDye Terminator v3.1 Cycle Sequencing Kit (Life Technologies) according to the manufacturer’s protocol. Sequencing products were analyzed on the ABI Prism 3100 Genetic Analyzer (Life Technologies).

## Results

A *BRCA1* causative founder variants were detected in 10 of the 158 (6.3%) unselected ovarian cancer cases. The c.5266dupC mutation was the most common, having been diagnosed in six patients, followed by the c.181 T > G mutation observed in three patients and the c.676delT mutation in one patient (Table 1). The median age of diagnosis of the 10 hereditary ovarian cancers patients was 55.5 years (range 41–82 years), compared with a median age of diagnosis of 58.75 years (range 22–84 years) for the 148 cases without a mutation. A mutation was found in 9.7% (3/31) of women diagnosed with ovarian cancer at or under the age of 50 compared to 5.5% (7/127) of women diagnosed at a later age. Among the 10 women with ovarian cancer and a *BRCA1* mutation, six reported a first- or second-degree relative with breast or ovarian cancer (60%). A mutation was present in 15.4% (6/39) of ovarian cancer patients with a positive family history and in 3.4% (4/119) of women with a negative family history. A significant family history, defined as a presence of first- or second-degree relative with breast or ovarian cancer, was observed in 24.7% (39/158) of patients with ovarian cancer (Table 2).

**Table 1:** The frequency of causative founder variants in unselected series of ovarian cancer patients

*BRCA1* causative variant	Number	Percent
c.5266dupC	6	3.8
c.181 T > G	3	1.9
c.676delT	1	0.6

**Table 2:** Prevalence of *BRCA1* causative variants in ovarian cancer by age of onset and family history

	Number of cases	Number with a mutation	Proportion with a mutation (%)
Age group			
≤ 50	31	3	9.7
> 50	127	7	5.5
Family history			
Positive	39	6	15.4
Negative	119	4	3.4
All cases together	158	10	6.3

## Discussion

The Podkarpacie region is located in the South-Eastern part of Poland, bordered by Ukraine and Slovakia. We found that 6.3% of unselected cases of ovarian cancer in this region carried one of 13 causative founder variants in the *BRCA1* or *BRCA2*. This is a lower rate than in other regions of Poland, where the frequency of *BRCA1* causative founder variants was observed in about 10–13.5% of ovarian cancer patients [2–8]. The frequency is, however, high enough to support genetic testing for all ovarian cancer patients in the region.

Since Podkarpacie is on a boundary region of Poland next to Ukraine, it is possible that the population slightly differs from other regions of Poland in terms of genetic makeup. In the nineteenth century, the region was part of Habsburg empire with significant minorities of Ukrainians and Jews, as well as a small proportion of Germans, Austrians, Hungarians and Armenians. Recently, Gorodetska et al. observed a slightly lower frequency of c.5266dupC and c.181 T > G *BRCA1* mutations in a series of ovarian cancer patients from Ukraine as compared to other Slavic populations [9–16]. Genetic testing of *BRCA1* mutations has been in place in the region since 2000. Several hundred mutation carriers have been diagnosed and a significant number of prophylactic oophorectomies have been performed. It may also, to some extent, affect the lower frequency of detected mutations than previously observed in other regions.

Our study was limited to screening for only 13 causative founder variants in *BRCA1/2* and possibly other mutations will be detected with whole *BRCA1/2* genes sequencing. In this study, the spectrum of detected *BRCA1* mutations was similar to the rest of Poland and to other Slavic countries [9–16]. We observed the *BRCA1* c.5266dupC mutation in 60% of carriers and the *BRCA1* c.181 T > G mutation in 30% of carriers. In one case, the *BRCA1* c.676delT mutation was detected. This mutation is rare in Polish population but should be included in the panel of recurrent mutations in the screening tests. Among the familial breast and ovarian cancers from this region, we also observed a higher frequency of the c.68_69delAG *BRCA1* mutation (data not shown), but this mutation was not observed in this series of consecutive ovarian cancers.

The mean age of diagnosis of the 10 cases with *BRCA1* mutations was 55.5 years, which is slightly higher than the mean age observed in other regions of Poland [6–8]. Possibly there are lifestyle/environmental factors which may influence later age of diagnosis, however, for non-carriers, the mean age of diagnosis was similar in Podkarpacie and in the rest of Poland (58.75 vs. 56.2–62.3 years) [6–8].

A strong breast or ovarian cancer family history was reported by 24.7% of ovarian cancer patients. It is significantly more frequent than observed by other authors [6, 8]. We think that this phenomenon may be caused by a larger number of family members than in other regions of Poland. Urbanization in this region used to be lower and fertility rate higher. We observed strong cancer family history in 6 out of 10 mutation carriers, which is more frequent than observed in other regions [6–8]. It can be explained by a relatively larger number of family members. However, between familial cases as well as between all patients with ovarian cancer we observed the lower frequency of mutation carriers, therefore we think that we could have missed some mutations while testing only causative founder variants.

There are several limitations to our study. The number of cases is relatively small and the results are based on 10 mutation-positive ovarian cancer cases. The increase of the study group was not possible because the registry of unselected, consecutive ovarian cancer patients was organized in 2013, and other hospitals did not create such a registry. Due to the high mortality of patients with ovarian cancer, a retrospective study based on a group diagnosed before 2013 may be biased. We screened only for the limited number of recurrent mutations and it is possible that other rare mutations were missed. Since the frequency of *BRCA*-carriers was significantly lower, including cases with family history of breast or ovarian cancer, than in other regions, the study based on sequencing of whole *BRCA1* and *BRCA2* should be performed.

## Conclusions

Approximately 6% of unselected ovarian cancer patients in the region of Podkarpacie Poland carry a *BRCA1* causative founder variants. These causative variants frequency was significantly lower than in other regions of Poland but it is sufficiently high to support the recommendation that all ovarian cancer patients undergo genetic testing regardless of the age of diagnosis or family history.

## Abbreviation

PCR: Polymerase chain reaction
